# Prader–Willi Syndrome in Adults: An Update On Nutritional Treatment and Pharmacological Approach

**DOI:** 10.1007/s13679-022-00478-w

**Published:** 2022-09-05

**Authors:** Luigi Barrea, Claudia Vetrani, Danilo Fintini, Giulia de Alteriis, Filippo Maria Panfili, Sarah Bocchini, Ludovica Verde, Annamaria Colao, Silvia Savastano, Giovanna Muscogiuri

**Affiliations:** 1 Dipartimento Di Scienze Umanistiche, Centro Direzionale, Università Telematica Pegaso, Via Porzioisola F2, 80143 Naples, Italy; 2 Department of Clinical Medicine and Surgery, Endocrinology Unit, Centro Italiano Per La Cura E Il Benessere del Paziente Con Obesità (C.I.B.O), University Medical School of Naples, Via Sergio Pansini 5, 80131 Naples, Italy; 3 Dipartimento Di Medicina Clinica E Chirurgia, Unit of Endocrinology, Federico II University Medical School of Naples, Via Sergio Pansini 5, 80131 Naples, Italy; 4 Endocrinology Unit, Bambino Gesù Children Hospital, Reference Center for Prader–Willi Syndrome, Rome, Italy; 5 School of Medicine, University of Rome Tor Vergata, Rome, Italy; 6 Cattedra Unesco “Educazione Alla Salute E Allo Sviluppo Sostenibile”, University Federico II, Naples, Italy

**Keywords:** Prader–Willi syndrome, Nutrition, Diet, Obesity, Drugs, Ketogenic diet, Nutritionist

## Abstract

**Purpose of Review:**

Prader–Willi syndrome (PWS) is a rare and complex genetic disorder with multiple effects on the metabolic, endocrine, and neurological systems, as well as behavioral and intellectual difficulties. Despite advances in understanding the genetic basis of obesity in PWS, there are conflicting data on its management. Therefore, the present manuscript aims to provide an update on the nutritional treatment and pharmacological approach in adult patients with PWS.

**Recent Findings:**

The management of obesity in patients with PWS is challenging and requires the cooperation of an experienced multidisciplinary team, including the nutritionist. An adequate clinical evaluation including nutritional and biochemical parameters should be performed to tailor the best therapeutic strategy. Both lifestyle and pharmacological interventions may represent useful strategies to prevent the high rate of morbidity and mortality related to PWS. The use of bariatric surgery is still controversial.

**Summary:**

Although it is imperative to adopt an obesity prevention strategy in childhood, there is promising evidence for the treatment of obesity in adulthood with current obesity medications in conjunction with lifestyle interventions

## Introduction

Prader–Willi syndrome (PWS), the most common syndromic form of childhood obesity, is due to the absent expression of genes located on the paternal chromosome 15q11.2-q13 [1]. The three main genetic subtypes are represented by paternal 15q11.2-q13 deletion, maternal uniparental disomy 15, and imprinting defect. Moreover, the methylation-specific multiplex ligation-dependent probe amplification analysis currently represents the test of choice for confirming the diagnosis of PWS in almost 99% of cases [2].

Patients with PWS are characterized by neonatal hypotonia, hyperphagia, developmental and cognitive delay, behavioral problems, dysmorphic features, and failure to thrive [3]. Notably, in patients with PWS, hyperphagia is associated with a lack of satiety leading to obesity and associated comorbidities [4]. In this context, nutritional intervention and behavioral modifications are among the most important factors in the treatment of patients with PWS to prevent obesity, type 2 diabetes (T2D), and cardiovascular diseases (CVD) [5]. Different dietary approaches were recommended for patients with PWS aiming to limit energy intake, including Mediterranean diet and Ketogenic diet [6, 7].

The childhood-onset obesity is also a trigger for severe complications during adult age, in particular body composition and endocrine alterations [8, 9], including growth hormone (GH) deficiency (GHD) [9], hypothyroidism [10], hypogonadism [11], and leptin resistance [12]. Of interest, GHD is present in 40–100% of the cases [9], and GH replacement therapy should be started as soon as possible because it can prevent obesity and improve psychomotor development [13]. In addition, patients with PWS have a higher incidence of metabolic complications, such as CVD, T2D, hypertension, and obstructive sleep apnea [5, 14, 15], thus contributing to increased morbidity and mortality in these patients [14, 16]. Insulin resistance is common and T2D is present in 7–24% of patients with PWS [17, 18]. In particular, T2D is poorly presented during childhood, while it is frequent in adult patients after the 5^th^ decade of life [17]. In this context, a collaboration between the nutritionist and the endocrinologist is essential to guarantee a strict follow-up of the patient, tailoring the therapeutic approach into the different stages of life.

Beyond nutritional approach, many drugs, including Metformin [19], Sibutramine, and Rimonabant [20, 21], Orlistat and Lorcaserin, and Naltrexone-Bupropion [22] have been used over the years in patients with PWS. Nevertheless, there is no solid evidence about their effectiveness and more extensive studies should be performed [19, 23]. Finally, although bariatric surgery is currently the most effective therapy to achieve weight loss in patients with very severe obesity, its use in patients with PWS remains still controversial [24].

Overall, although steps have been taken in understanding the genetic basis of obesity in PWS, there are still some contradictory data on its management. Therefore, the present manuscript aims to provide an update on the nutritional management and pharmacological approach in adult patients with PWS.

## Obesity and Comorbidities in Patients With PWS

The clinical picture is complex and varies considerably across the life stages: axial hypotonia, inability to suck, and hypothermia are common at birth, and often require prolonged incubation and use of nasogastric or gastric tube to provide adequate nutrition and to avoid short stature and/or decreased growth velocity [9]. Children with PWS have an impairment of central nervous system networks, including hypothalamic dysfunctions, which involve both the hunger/satiety circuitry and the main endocrine axes, and are also associated with temperature instability, high pain threshold, and aberrant sleep cycle [25]. In addition to short stature, the developmental delay and cognitive disability are typical features which tend to worsen over time, leading to an autism-like phenotype, including self-mutilation behaviors, skin picking, learning problems, irritability, and compulsive behaviors [26].

The abnormal attitude towards food has always been considered one of the peculiar traits of the patients with PWS affecting all stages of life, although in different ways [9, 16]. In fact, several nutritional and growth phases can be identified in patients with PWS up to the development of the typical chronic hyperphagia that characterizes the syndrome [27]. The nutritional phase 0, which concerns intrauterine life, is characterized by low birth weight and length, with decrease fetal movements. Up to about the first 3 months of life (nutritional phase 1a), PWS newborn is hypotonic, manifests feeding and suckling deficits, and feeding via nasogastric or gastric tube is often required to ensure sufficient nutrition—afterwards up to 12–18 months, (nutritional phase 1b) the feeding improves, and the infants begin to exhibit normal appetite and growth [27]. After 18–24 months of age, the child begins to gain weight: initially without a change in food intake or interest in food (nutritional phase 2a), while around 3–4 years a relative hyperphagia appears (nutritional phase 2b), with a growing interest in the food [27]. A few years later in childhood (nutritional phase 3), the classical hyperphagia occurs, characterized by constant hunger, lack of satiety, irritability if food is denied, and a range of dysfunctional food-related behaviors, including theft, nighttime eating, and consumption of expired, spoiled or frozen food [27]. If the access to food is not controlled by tailored dietary interventions and behavioral management during childhood, severe obesity develops, triggering increased morbidity and mortality later in adulthood [14, 16]. Thus, the prevalence of obesity in patients with PWS varies according to age, from 40% in pediatric subjects and up to 82–98% in adults [24, 28]. However, beyond weight gain, obesity involves a series of obesity-related diseases, which in patients with PWS can have a stronger impact, considering the early onset of obesity, the difficulties in adhering to lifestyle changes, use of psychotropic medications, and the inability to communicate some symptoms linked to mental retardation [24, 28]. The prevalence of T2D in patients with PWS ranges 7–24%, with lower rates in childhood and higher rates in adults with obesity after the 5^th^ decade [17, 18]. However, some aspects related to the pathogenesis of altered glucose metabolism and T2D and are still matter of debate. Indeed, besides a familial component in insulin resistance, as in general population, patients with PWS have fairly low fasting insulin concentrations and a lower incidence of insulin resistance compared to that expected considering the body mass index (BMI), probably due to a greater distribution of adipose tissue in the subcutaneous than in visceral site [15, 29, 30]. In any case, during the entire life span of patients with PWS, glucose metabolism must be carefully monitored annually by measuring glycated hemoglobin (HbA1c), fasting and post-load glucose, and approaching diabetes management using similar pharmacological agents according to the guidelines for the general population [15]. The glucose profile must also be assessed in patients undergoing a replacement therapy with GH and/or sex steroids, due to possible diabetogenic effects [15].

Cardiovascular diseases are one of the main causes of death in patients with PWS [31]. A nationwide, population-based cohort study carried out in Denmark including 155 patients with PWS that were followed-up from birth through to first occurrence of an outcome of interest found an increased risk of myocardial infarction, deep venous thrombosis and pulmonary embolisms in these subjects compared to a matched population cohort [32]. Therefore, monitoring cardiovascular risk factors starting as early as during adolescence is essential, evaluating at least annually electrocardiogram, lipid, and pressure profile, carrying out second level investigations, such as cardiac ultrasound, 24 h ambulatory blood pressure monitoring, or inducible myocardial ischemia test when necessary [33, 34]. However, as for glucose metabolism, no significant differences in lipid profile (triglycerides, total cholesterol, HDL and LDL cholesterol) were detected between patients with PWS and BMI-matched patients without PWS, likely because patients with PWS have less visceral fat content and this could preserve lipid profile from the detrimental effect of visceral fat excess [35, 36]. In a cross-sectional study on 109 children with PWS aged 2–18 years (50 with obesity and 59 without obesity), and 96 controls with simple obesity matched for age, gender and BMI, it was shown that children with PWS without obesity showed significantly lower frequency of hypertension (12%) than subjects with PWS and obesity (32%), and controls with simple obesity (35%), suggesting the crucial role of obesity rather than the syndrome “per se” in the absence of excessive weight [37].

The impact of modern interventions on mortality risk was evaluated by the PWS Association 40-year mortality syndrome-specific database [38]. In this cohort study, Manzardo and colleagues examine the survival trends in patients with PWS using death reports from years 2000 to 2015. This study demonstrated that, although the respiratory failure was as a leading contributor to mortality in patients with PWS, the survival estimates for patients with PWS have increased since 2000, especially for fatal cardiac events in females and thrombotic and gastrointestinal-related mortality, most likely for earlier diagnosis and proactive interventions to prevent very severe obesity [38]. Therefore, this study highlighted how an early and timely lifestyle intervention aiming to limit weight gain is essential to improve life expectancy and reduce mortality in patients with PWS.

## Etiological Mechanisms of Weight Gain in Patients with PWS

Multiple alterations in energy homeostasis are commonly reported in patients with PWS and contribute to the massive weight gain in these patients [24]. In particular, patients with PWS presented a chronic imbalance between higher energy intake, due to hyperphagia [9], and lower total and resting energy expenditure measured by indirect calorimetry when compared with subjects with age-, sex-, and BMI-matched controls [6]. The mechanism for the significant overall reduction in energy expenditure in patients with PWS is multifactorial. It includes hypotonia and abnormal body composition, decreased lean body mass and increased fat mass (FM) [39], multiple pituitary hormone deficiencies, and low physical activity [8]. In this context, tailored dietary energy recommendations are crucial for the management of patients with PWS. In addition, it was observed that in patients with PWS the consumption of the recommended daily allowance of calories for age, gender and height does not guarantee the maintenance of body weight, but determines a weight gain [27].

Hypothyroidism is diagnosed in patients with PWS with a prevalence ranging 4–72% [40, 41]. Hypothyroidism, that contributes to the reduction of the basal metabolic rate [10], can be primitive or depend on hypothalamic-pituitary dysfunction. In the latter case, it results in low or low-normal concentrations of thyroid-stimulating hormone (TSH) and low concentrations of free thyroxine. Therefore, early screening of TSH and free thyroxin concentrations is recommended, and hypothyroid patients are treated with levothyroxine at standard replacement doses [42]. Additionally, thyroid function should be monitored 3–4 months after the start of GH therapy since GH could be responsible of an increased conversion of T4 to T3 [43].

GHD is the most frequent and studied endocrinopathies in patients with PWS, with a prevalence ranging from 40 to 100% [9]. In particular, the GH 24 h secretion is decreased, along with the reduction of insulin-like growth factor I (IGF-I) production and a lower response of GH to stimulation tests [44]. To start GH replacement therapy, the diagnosis of patients with PWS without any confirmatory test is sufficient, while it is necessary to perform dynamic tests after the attainment of final height as the deficit is often not confirmed in adulthood [13]. GHD also contributes to the alteration of body composition and weight gain, with decreased lean mass, increased FM, mainly truncal FM with increase waist/hip ratio, scarce muscle tone and strength, reduced energy expenditure, and exercise tolerance [45–47]. These alterations significantly improve with GH replacement therapy with reduction in BMI and FM in both children and adults with PWS [45–47]. Instead, evidence on patients for 12–24 months after GH replacement therapy shows a progressive increase in BMI, especially visceral adipose tissue [48]. To prevent the onset of obesity and to improve psychomotor development, it is advisable to start GH therapy as soon as possible, between 3 and 6 months of age, monitoring the glucose profile due to the diabetogenic effect of the GH, and the risk of OSA by polysomnography [13]. The recommended starting dosage is 0.5 mg/m^2^/day in children and 0.1–0.2 mg/day in adults by monitoring the IGF-I values, which should be maintained within the upper half of the reference range [13].

**Fig. 1:**
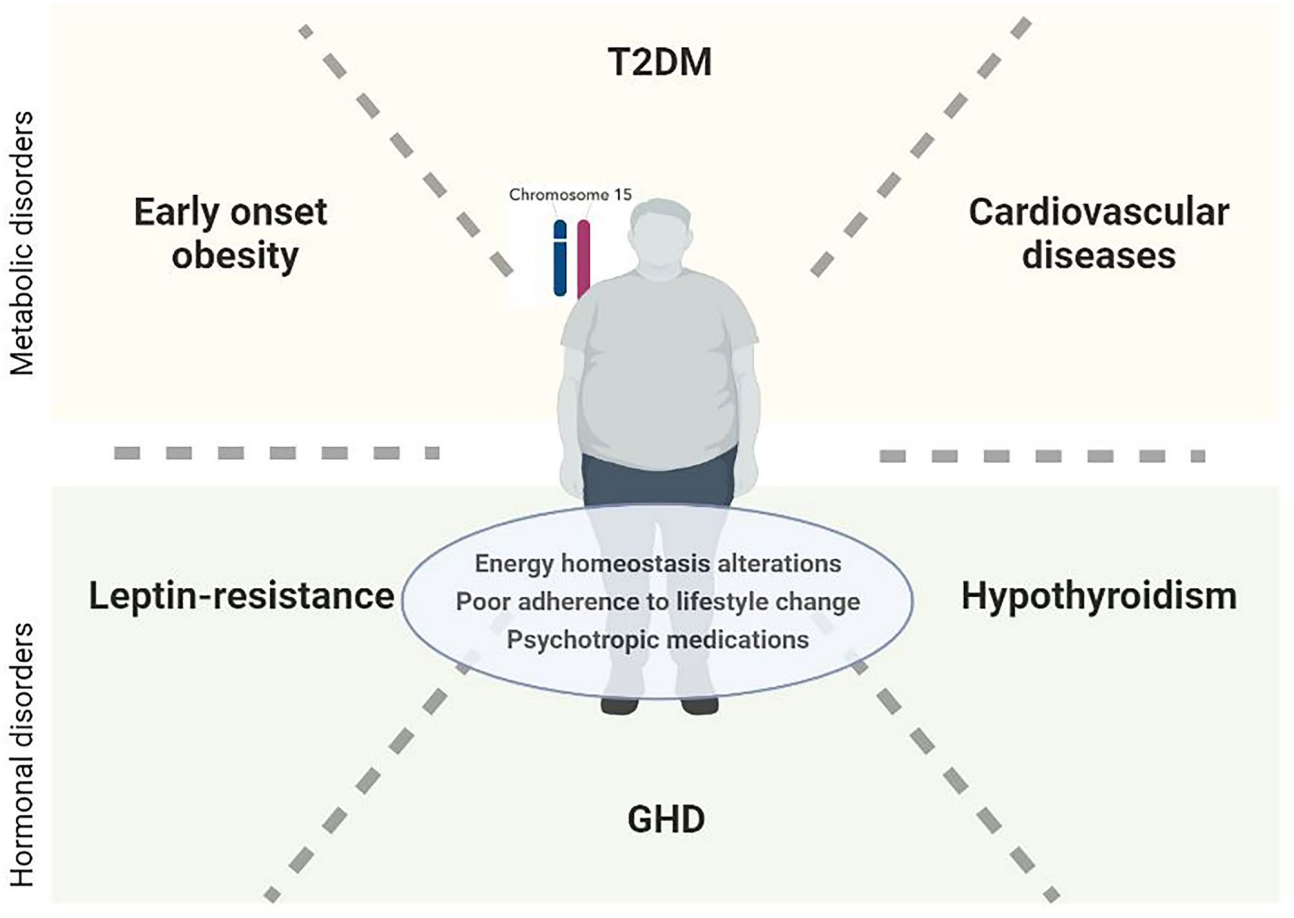
Metabolic and hormonal disorders in patients with PWS. T2DM, type 2 diabetes mellitus; GHD, growth hormone deficiency

Another mechanism potentially involved in weight gain could be the leptin resistance [49]. In a PWS mouse model, a progressive central leptin insensitivity has been observed and it predicted the reduction in the anorexic leptin-mediated effect and the energy expenditure, likely through the impairment of the activation of leptin-responsive pro-opiomelanocortin neurons and the release of the melanocortin receptor agonist α-melanocyte-stimulating hormone [12]. Therefore, weight gain tends to worsen the leptin resistance, which in turn increases hyperphagia and reduces the energy expenditure by amplifying the phenomenon in a vicious downhill cycle of weight gain [12] (Fig. 1).

Physical activity plays a pivotal role in the energy balance in patients with PWS [50]. Several studies indicate that both children and adults with PWS perform less physical activity than age-matched controls without PWS [51, 52]. According to the recommendations for the management of patients with PWS, physical exercise should be an important part of patients’ daily life and prescribed as a coadjutant treatment to GH therapy, in addition to caloric restriction [33]. However, the achievement of recommended level of moderate-intensity physical activity is challenging for patients with PWS [53]. A recent systematic review of 22 studies reported that patients with PWS (n = 356) present a decreased physical performance and impaired cardiorespiratory and hormonal responses to exercise [54]. Most long-term exercise interventions have proven to decrease FM while improving physical performance, along with significant improvement in cardiorespiratory fitness and increased muscle strength [55, 56]. Some benefits have also been reported in adults with PWS and T2D with reduction in HbA1c concentrations and total daily doses of insulin or oral hypoglycemic agents, and also on biomechanical variables, including improvement in coordination, strength, agility, and hand grip strength [57]. Furthermore, no adverse events have been found following physical activity programs in patients with PWS, although continuous supervision and support from experienced personnel is required. Very recently, a systematic review of controlled trials including 25 studies evaluated the effectiveness of physical activity interventions in patients with PWS and confirmed that both total volume of physical activity and physical exercise-related energy expenditure were lower in patients with PWS compared to patients with non-syndromic obesity [50]. Although no significant effect on weight and FM in children with PWS was observed, habitual physical activity programs were positively associated with lean body mass and bone parameters, and with improved physical function, not only in terms of muscle strength and walking distance, but also as locomotors coordination.

## Assessment of Nutritional Status in Adult Patients with PWS

The evaluation of nutritional status represents a pillar in the management of patients with PWS. It consists in the assessment of dietary intake and the evaluation of body composition, which provide important information to develop tailored interventions in these patients.

The assessment of dietary intake allows the estimation of energy, macronutrients, and other dietary components provided by the usual diet and the adequacy of the diet and potential nutritional deficiencies [58]. Different methods can be used for this purpose (24 h recall, dietary record, food frequency questionnaire, dietary history), but the choice depends on patients’ compliance [59]. On the other hand, the assessment of body composition allows a more reliable and objective measurement of the nutritional status. Moreover, the assessment of body composition can be used to monitor the effect of nutritional interventions or disease-related data (clinical features as well as prognostic information) [60]. Several methods can be used to evaluate the body composition with different outcomes on precision and accuracy [60]. Anthropometry is the easiest method to detect information on nutritional status [60]. Body weight and height can be used to calculate BMI and provide an estimation of cardiometabolic risk linked to overweight/obesity. Indeed, BMI > 25.0 kg/m^2^ has associated with shorter longevity and increased risk of cardiovascular morbidity and mortality [61]. In addition, the assessment of waist circumference can give information about abdominal adiposity, which represents a risk factor for CVD and T2D [60, 62]. Nevertheless, it is important to underline that anthropometric measurements need standardized protocols to increase the accuracy and precision of collected data [63]*.*

Imaging techniques (dual-energy x-ray absorptiometry, computed tomography, magnetic resonance, and ultrasound scanning) are the most advanced and valid methods to detect body composition. However, these techniques are expensive, time-consuming, or expose patients to radiation [64]. Therefore, bioelectrical impedance analysis [65] has been widely used in clinical practice and in research studies to assess body composition. Although BIA does not directly measure body composition, it provides information about FM, free fat mass (FFM) [39], and body water (total, intracellular and extracellular water) [66]. As for the evaluation of nutritional status in adults with PWS, only limited evidence is available on dietary habits in these patients, and studies were carried out in small-size cohorts (n < 20 participants). In particular, these cross-sectional studies showed that adults with PWS might present inadequate intake of some nutritional components (i.e., dietary fibers, vitamin D, and calcium) [67, 68]. Martinez Michel and colleagues provided an overview of some specific food behaviors in patients with PWS [69]. This review of 27 studies suggested that patients with PWS prefer sweet tastes and calorie-dense foods that might explain, at least in part, the increased body weight. Nevertheless, these findings should be confirmed in future studies with a larger sample size. Conversely, more evidence is available on the body composition in patients with PWS and it will be discussed in detail in the following paragraph.

### Body Composition Assessment

Several studies in children and adolescents undergoing dual-energy X-ray absorptiometry (DXA) have shown that patients with PWS presented a higher FM and a lower FFM than control individuals with simple obesity [6]. As mentioned above, DXA is the best approach to assess body composition, but it is not a feasible tool to be used in the clinical practice for the management of patients with PWS [6]. Indeed, these patients might need a strict follow-up to assess the effect of therapeutic interventions and evaluate their cardiometabolic risk. Therefore, most recent studies focused on the assessment of body composition by BIA in children [70], as well as adult patients with PWS [70, 71]. Notably, Bedogni and colleagues evaluated the body composition both by BIA and DXA in a group of 27 women with PWS (age: 30 ± 3 years, BMI 41.5 ± 4 kg/m^2^) and 54 control women matched for age- and BMI (age: 31 ± 2 years, BMI 41.8 ± 2 kg/m^2^) [70]. The results showed that women with PWS had lower FFM than control women (44.4 ± 2% *vs*. 49.0 ± 1%, respectively) suggesting that individuals with PWS might require population-specific equations to the prediction of body composition by BIA [70]. The same research group extended this finding and confirmed lower FFM in 34 women with and 21 men with PWS and obesity (46.8 ± 6% and 49.5 ± 6, respectively) as compared to reference values [70]. The reduction of FFM might explain, at least in part, the reduced energy expenditure observed in patients with PWS [72, 73]. Indeed, total energy expenditure (TEE) consist in four different components: resting energy expenditure (REE) [36], activity energy expenditure [74], sleeping energy expenditure (SEE), and diet-induced thermogenesis (DIT) [75].

A systematic review including 10 studies in humans reported that patients with PWS have lower REE and AEE values than individuals with non-syndromic obesity, with no major changes in the other components of TEE (SEE and DIT) [6]. As REE and AEE are strictly related to the amount of FFM, mainly muscle mass, improving FFM in patients with PWS may represent a useful strategy to increase TEE [6]. REE accounts for 70–80% of daily TEE and, therefore, it is calculated for the estimation of dietary energy requirement in clinical practice [6]. It is to underling that current equations for the prediction of REE consider only age, sex, height, and BMI, and might overestimate REE in adults with PWS [73, 76]. As an example, a study in 80 patients with PWS (age: 17–50 years, BMI 39.1 kg/m^2^) showed that using Harris–Benedict equation, one of the most used prediction equations in clinical practice, there was an overestimation of REE > 7% [73]. Therefore, according to the peculiar features of patients with PWS (low FFM and low TEE) new prediction equations have been validated for the calculation of REE (also known as basal metabolic rate, expressed as MJ). The equations proposed by Lazzer and colleagues are: 1) body mass [77] × 0.052 + sex (1 for males and 0 for females) × 0.778 + age (years) × 0.033 + 2.839; or 2) FFM [77] × 0.074 + FM [77] × 0.042 + sex (1 for males and 0 for females) × 0.636 – age (years) × 0.037 + 2.515), which also consider body composition (FFM and FM assessed by BIA) [73]. Besides FFM and FM, phase angle [78] is a BIA-derived measure that provides information on cellular health and integrity [66]. In addition, PhA has been appointed as a reliable tool to detect inflammatory status in many diseases [79–81]. Increased plasma concentrations of inflammatory indices have been detected also in patients with PWS [82–84]. Interestingly, a cross-sectional study investigated the relationship between PhA and inflammation (measured by C-reactive protein, CRP) in 15 patients with PWS (28 ± 6.8 years, 43.8 ± 10.7 kg/m^2^) compared to gender-, age-, and BMI- matched individuals without PWS (n = 15, 30 ± 6.9 years, 43.9 ± 8.8 kg/m^2^) [80]. Patients with PWS presented lower PhA and higher plasma concentrations of CRP than control individuals. Moreover, these two parameters were inversely associated also after adjustment for the main confounding factors (gender, BMI, and waist circumference) [80]. These findings suggest that PhA could be a useful non-invasive marker of inflammation to consider in the management of patients with PWS [80].

PWS, Prader–Willi syndrome; BMI, body mass index; SD, standard deviation; GLP-1, glucagon-like peptide-1; GIP, gastric inhibitory polypeptide; GLP-2, glucagon-like peptide-2; FFA, free fatty acids; TG, triglycerides; HDL, high-density lipoprotein; CI, confidence interval; LDL, low-density lipoprotein.

### Nutritional Approach

A relevant aspect in the management of the patients with PWS is the adherence to a diet starting from the first months of life to favor a regular body growth and, subsequently, to cope with the incipient hyperphagia and to prevent or treat the excess of weight throughout the life span [6, 7, 89]. Therefore, the nutritionist represents together with the endocrinologist the key figures in the management of patients with PWS, since a close nutritional follow-up, adapted to the patient's life stages and in agreement with the caregivers is essential. A low-calorie diet is the standard recommendation for patients with PWS to achieve weight management, and several types of diets have been suggested, i.e., a low-fat low-calorie diet, a modified plant-based food pyramid, and the ‘red, yellow, green’ diet, which prefers plant foods restricting high-fat and high-energy ones [90, 91]. However, all the proposed diets aimed to restrict fats and energy and offer no specific recommendations about the amount of the other nutrients and fiber intake.

Currently the most promising strategy seems to be a well-balanced low-calorie diet. In a study on 63 patients with PWS, it was demonstrated that a balanced energy-restricted diet of approximately 30% fat, 45% carbohydrates (at least 20 g of fiber/day), and 25% protein significantly improved body weight composition in patients with PWS compared to a standard energy-restricted diet [85]. In addition, in the context of a balanced low-calorie diet, it is recommended to consume small and split meals throughout the day, including snacks, to control hyperphagia and to avoid repeated requests for food, theft, and obsessive behavior towards food that could worsen during more drastic diets [85].

As for ketogenic diet, a study in eight hospitalized patients with PWS (age 9–18 years) showed that a low-carbohydrate high-fat diet (15% carbohydrate, 65% fat, and 20% protein) increases glucagon-like peptide-1 (GLP-1) and reduces ghrelin/GLP-1 ratio, possibly limiting food intake and improving glycemic control as compared to a low-fat high-carbohydrate diet (65% carbohydrate, 15% fat, and 20% protein) [86]. Other potential benefits of carbohydrate restriction may include fat mobilization and oxidation, and reduction in the triglycerides/HDL ratio, a marker of insulin resistance. However, ketogenic diet may increase CRP and liver enzyme, and therefore, longer-term studies are needed to confirm its efficacy and safety, and to recommend its use only in selected patients and under strict medical supervision [86]. Accordingly, a clinical feasibility study investigated the effects of a 4 month-ketogenic-like diet (i.e., Modified Atkin Diet, i.e., 10–15 g of net carbohydrate) in a very small group of children with PWS (n = 4, 6–12 years) [87]. One patient lost 2.9 kg; the others maintained their weight. Nevertheless, positive effects on hyperphagia as well as on behaviors were reported by parents and relatives [87].

Therefore, even though results deriving from the use of ketogenic diet in patients with PWS populations are promising, no conclusions can be drawn on the effectiveness and safety of this diet, due to small sample size and short-term duration of the studies.

On the other hand, in a recent study adults with PWS (N = 45, median age of 26 years) underwent a six years-program based on Mediterranean diet and physical activity [88]. More in details, participants underwent a 3-weeks-metabolic rehabilitation program which was performed at the beginning (baseline) and after three and six years. In between, the patients were followed every six months [88]. The mean weight loss was 3.6 and 4.6 kg after three and six years, respectively. FM decreased by 2.3% and 1.8% after three and six years, respectively. In addition, total and LDL cholesterol concentrations were significantly lower after six years (-11.7 and 8.1 mg/dl, respectively) [88].

## Pharmacological Approach in Patients with PWS

Besides nutritional management, GH replacement therapy in PWS children with GHD has demonstrated to decrease FM, increase FFM, and improve both motor and mental performance [92]. Nevertheless, GH therapy is not effective in the reduction of PWS-specific hyperphagia and in the long-term body weight control [93].

Over the years, several anti-obesity drugs have been used to support nutritional therapy in patients with PWS [31, 49]. Orlistat is a gastrointestinal lipase inhibitor that limits fat absorption to up to 30% of ingested fats without exerting central nervous system effects. It has demonstrated only modest efficacy in patients with PWS, likely due to poor compliance for gastrointestinal side effects [94]. Metformin is an oral hypoglycemic drug used for the management of T2D and pre-diabetes in individuals with obesity. A pilot study with metformin supplementation in 21 children and adolescents with PWS showed an improvement of the food-related distress and anxiety, evaluated by hyperphagia questionnaire, but no effects on body weight [19]. Similar effects were observed also after Topiramate supplementation, an antiepileptic drug used also in the treatment of atypical psychoses that acts as modulator on Na^+^ channels, gamma-aminobutyric acid (GABA) concentrations, and α-amino-3-hydroxy-5-methyl-4-isoxazolepropionic acid (AMPA) and kainate receptors. An 8-week double-blind randomized placebo-controlled trial in 62 patients with PWS demonstrated an improvement of hyperphagia—evaluated as behavior and severity scores by Dykens Hyperphagia Questionnaire—after Topiramate group *versus* placebo group, with no effects on BMI [95]. Therefore, both metformin and topiramate may play a role in the behavioral control of hyperphagia but without contributing to weight loss.

Other promising drugs to reduce appetite and increase energy expenditure in patients with PWS were Sibutramine, an unspecific inhibitor of serotonin and norepinephrine reuptake, and Rimonabant, an endocannabinoid CB1 receptor antagonist. Nevertheless, these drugs were withdrawn from the market for their serious cardiovascular and psychiatric adverse events registered during clinical trials [20, 21].

Naltrexone-Bupropion is a combination of a mild inhibitor of dopamine and norepinephrine reuptake (Bupropion) with an agonist of the μ-opioid receptor (Naltrexone) that acts synergistically to activate pro-opiomelanocortin (POMC) neurons in the hypothalamic, in particular in the neurons in the arcuate nucleus (ARC), resulting in appetite suppression [96, 97]. Until now the effectiveness of this association for 6 months has only been described in a case report in a girl with PWS, showing an improvement of eating habits, without a significant reduction of BMI [22].

Considering the multiple alterations in the endocrine control of eating behavior in patients with PWS that involve both central and peripheral signals of hunger-satiety network, a number of studies have been performed to modulate hyperphagia [31]. In particular, Bueno and colleagues classified patients with PWS according to their fasting and postprandial cluster of a hormones to tailor more specific drugs development. They highlighted that most of patients with PWS present a similar altered endocrine profile, characterized by high concentrations of ghrelin, leptin, peptide YY, gastric inhibitory polypeptide, and GLP-1 [98]. Accordingly, GLP-1 receptor agonists may act determining a decrease in appetite and weight and stimulating the glucose-dependent insulin secretion with protective effects on pancreatic β-cells and cardiovascular system [99]. A six-month treatment with exenatide, a short-acting GLP-1R agonists, was evaluated in a longitudinal study on 10 overweight/obese patients with PWS—3 patients were affected by T2D—showing a reduction in appetite scores and an improvement in HbA1c, with no changes in weight or BMI [100]. In addition, in a report of six cases of patients with PWS and T2D never treated with GH, the treatment with 1.2 to 1.8 mg/day of liraglutide, a long-acting GLP-1R agonists, or 20 mg/day of exenatide for two years, a trend to reduced HbA1c and mean blood glucose, BMI, and waist circumference [23]. Although clinical trials with GLP-1 agonists are still ongoing, these findings suggested that GLP-1 receptor agonists could represent a useful tool in the management of patients with PWS, with promising effects on hyperphagia and body weight [49].

On the other hand, alterations in the oxytocinergic system have been described in patients with PWS, which have been linked to some of clinical features of patients with PWS, such as hyperphagia, obesity, and social behavior disorders [49]. Preliminary studies in both adolescents and adults with PWS suggested a beneficial effect of nasal oxytocin administration in improving social behavior. A randomized, double-blind phase 2 study confirmed the efficacy of nasal oxytocin administration in infants on oral feeding skills and social behavior. Moreover, a randomized, double-blind trial with intranasal carbetocin, an oxytocin receptor selective compound, in adolescents with PWS showed an improvement in hyperphagia score and behavioral symptoms after 14 days of treatment [49].

As above mentioned, due to ghrelin orexigenic action, hyperghrelinemia has been suggested as a potential cause of increased appetite and weight gain in patients with PWS [49]. A European multicentric randomized placebo-controlled study evaluated the effectiveness of livoletide (AZP-531), a non-acylated ghrelin analog, in patients with PWS [101]. The daily injection of a dose of AZP-531 for 14 days in 47 patients with PWS was effective in reducing the hyperphagia score, waist circumference and FM, without inducing serious adverse effects. However, no significant changes in ghrelin concentrations were observed, and all beneficial effects were not maintained after three months of treatment [102].

## Surgical Approach in Patients with PWS

A further therapeutic tool to consider in patients with PWS is bariatric surgery [24, 103]. However, current data concerning bariatric surgery effectiveness in genetic and syndromic obesity, specifically in patients with PWS, are generally based on few case reports, and results are not easy to compare due to differences in surgical procedures and duration of follow-up [49].

A retrospective analysis of 60 patients with PWS [104] showed that weight loss at 5 years after surgery is significantly lower in individuals with PWS than in individuals with non-syndromic obesity, with a relatively high post-surgical complication rate. However, the results of this study are difficult to interpret due to the heterogenicity of bariatric procedures and the high dropout rate at follow-up (at 5 years only 11 patients are reported). In addition, the high incidence of postoperative issues was likely due to the inclusion of obsolete bariatric surgery procedures, as well as some patients with PWS-specific conditions (i.e., abnormal pain threshold, inability to vomit, predisposition to acute gastric dilatation, or more severe preoperative clinical conditions).

Among surgical procedures, sleeve gastrectomy and mini gastric bypass (MGB) seem to be the better choices in patients with PWS considering both the effectiveness in the achievement and maintenance of weight loss and the reduced incidence of surgical complications. In particular, in three young male patients with PWS (mean age 15.6 years), laparoscopic MGB appeared effective in inducing a significant and stable weight loss (79% weight loss two years after surgery), without relevant nutritional deficiencies, weight regain, or need for revision surgery [65]. In a Chinese study, three patients with PWS underwent bariatric surgery, two-sleeve gastrectomy and one MGB [105]. After a median follow-up of 33 months (range 24–36 months), mean weight loss and percentage of weight loss at 2 years were 32.5 kg (24.9–38.3 kg) and 63.2% (range 50.5–86.2%), respectively, without major postoperative complications. Moreover, sleeve gastrectomy performed in 24 children and adolescents with PWS (average age 10.7 years) resulted in 14.7% weight loss at 1 year (n = 22) and 10.7% (n = 7) at 5 years, as compared to adolescents with non-syndromic obesity, and without major postoperative complications [106].

Results of bariatric surgery in 24 children/adolescents PWS compared to 72 non-PWS subjects matched for age, gender and BMI, undergoing a laparoscopic sleeve gastrectomy, have recently been published [107]. In the patients with PWS, the decrease in BMI was 15 kg/m^2^ at 1 year and 11 kg/m^2^ at 5 years, as in the non-PWS group. Moreover, a 81.8% of comorbidities remission was observed in particular for OSA [107]. Conversely, a study including five patients with a 10-year follow-up did not report sustainable long-term weight loss or comorbidity resolution after [108].

It is to underline that intellectual disabilities raise relevant ethical concerns in the bariatric treatment of adolescents with PWS and require an accurate preoperative analysis of psychological, social, and ethical aspects to adequately support patients and parents, and the long-term monitoring of the surgical outcome [109].

Therefore, bariatric surgery in patients with PWS remains still controversial and requires the collaboration of an expert multidisciplinary team and the development of more innovative bariatric treatment plans tailored to patients with PWS to obtain effective and stable weight loss, and to reduce severe health complications in these patients Table 1.

## Conclusion

**Fig. 2:**
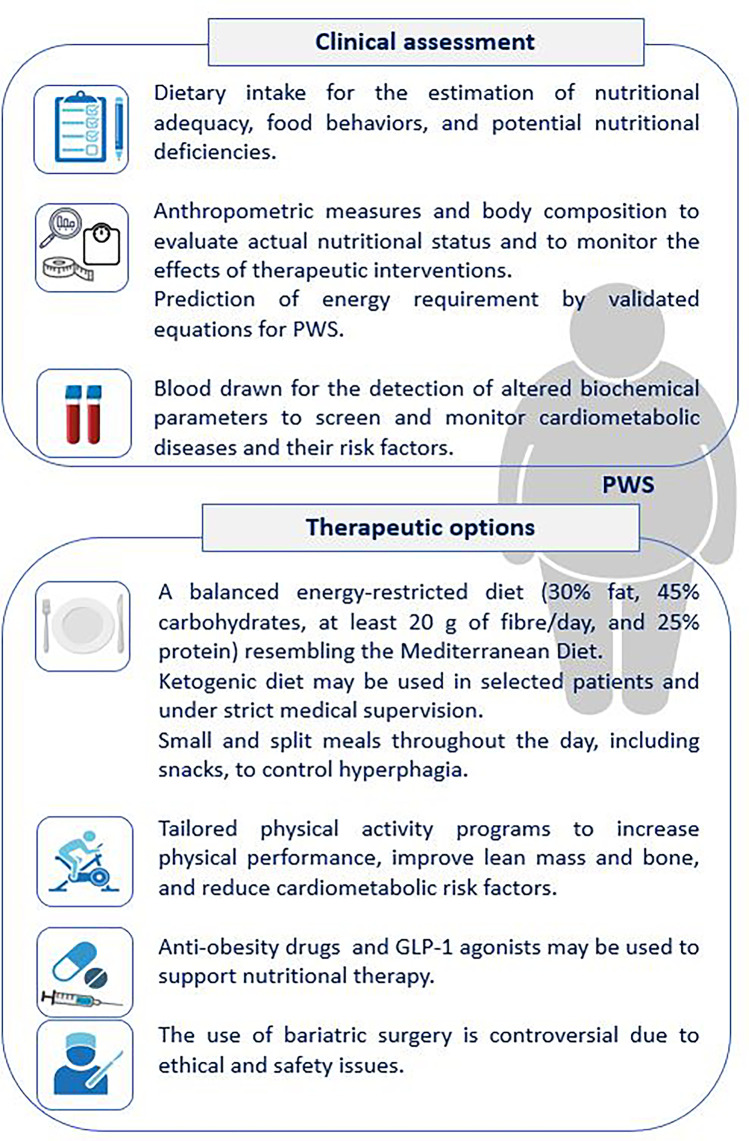
Clinical assessment and potential therapeutic strategies for the management of obesity and its complications in adults with PWS. Abbreviations: PWS, Prader–Willi syndrome; GLP-1, glucagon-like peptide-1

PWS is a complex disorder, and this syndromic obesity predisposes to an early risk of developing several health conditions as cardiometabolic diseases. Therefore, the management of obesity in patients with PWS is challenging and requires the cooperation of an experienced multidisciplinary team, including the nutritionist. An adequate clinical evaluation including nutritional and biochemical parameters should be performed to tailor the best therapeutic strategy (Fig. 2). Both lifestyle and pharmacological interventions may represent useful strategies to prevent the high rate of morbidity and mortality related to PWS. The use of bariatric surgery is still controversial (Fig. 2). Nevertheless, further studies are needed to pave the way to new therapeutic scenarios in the management of obesity in PWS.

**Table 1:** Significant studies on diet in patients with Prader–Willi syndrome

Reference	Type of study	Subjects	Diet	Control diet	Main results
Miller et al*. *[85]	Longitudinal study	Sixty-three children with PWS, aged 2–10 years	Well-balanced, energy-restricted diet (30% fat, 45% carbohydrates and 25% protein, with at least 20 g of fiber/day)	Energy-restricted only diet (10–23% fat, 50–70% carbohydrates and 15–20% protein, with 12 g or less of fiber/day)	Those who followed the well-balanced, energy-restricted diet had lower body fat (19.8% vs 41.9%; p < 0.001) and weight management (BMI SD score 0.3 vs 2.23; p < 0.001) than those who followed the energy intake recommendations but did not alter the macronutrient composition of the diet
Irizarry et al*. *[86]	Longitudinal crossover study	Eight children with PWS, aged 9–18 years	Low-carbohydrate high-fat diet (15% carbohydrate, 65% fat, and 20% protein)	Low-fat high-carbohydrate diet (65% carbohydrate, 15% fat, and 20% protein)	Compared to low-fat high-carbohydrate diet low-carbohydrate high-fat diet: reduced postprandial insulin concentrations (p = 0.02), increased fasting GLP-1 and GIP concentrations and increased postprandial GLP-1 (p < 0.02); reduced fasting ghrelin/GLP‐1 ratio (p = 0.0078); increased FFA and fatty acid oxidation (p < 0.001); reduced fasting TG and TG/HDL ratio (p < 0.01); increased concentrations of branch chain amino acids (p < 0.01)
Felix et al*. *[87]	Clinical feasibility study	Four children with PWS, aged 6–12 years	Modified Atkin Diet (10–15 g of net carbohydrate, protein and fat on individual basis)	n/a	One patient lost 2.9 kg; the others maintained their weight. Positive effects on hyperphagia as well as on behaviors were reported by parents and relatives
Bedogni et al*. *[88]	Retrospective cohort study	Forty-five adults with PWS and obesity, aged 22–30 years	Hypocaloric Mediterranean diet	n/a	The mean weight change was -3.6 ( p = 0.08) Kg at 3 years and − 4.6 (p = 0.02) Kg at 6 years, and that of BMI was -1.7 (p = 0.06) kg/m^2^ at 3 years and -2.1 (p = 0.02) kg/m^2^ at 6 years. A decrease of about 2% in fat mass per unit of body mass was observed. A possibly clinically relevant decrease in total and LDL cholesterol was also observed

## References

[1] Angulo MA, Butler MG, Cataletto ME (2015). Prader-Willi syndrome: a review of clinical, genetic, and endocrine findings. J Endocrinol Invest.

[2] Cassidy SB, Schwartz S, Miller JL, Driscoll DJ (2012). Prader-Willi syndrome. Genet Med.

[3] Mackenzie ML, Triador L, Gill JK (2018). Dietary intake in youth with prader-willi syndrome. Am J Med Genet A.

[4] Guinovart M, Coronas R, Caixas A (2019). Psychopathological disorders in Prader-Willi syndrome. Endocrinol Diabetes Nutr (Engl Ed).

[5] Muscogiuri G, Barrea L, Faggiano F (2021). Obesity in Prader-Willi syndrome: physiopathological mechanisms, nutritional and pharmacological approaches. J Endocrinol Invest.

[6] Alsaif M, Elliot SA, MacKenzie ML, Prado CM, Field CJ, Haqq AM (2017). Energy Metabolism Profile in Individuals with Prader-Willi Syndrome and Implications for Clinical Management: A Systematic Review. Adv Nutr.

[7] Krasinska A, Skowronska B. Prader-Willi Syndrome - nutritional management in children, adolescents and adults. Pediatr Endocrinol Diabetes Metab. 2017;23(2):101–106. 10.18544/PEDM-23.02.0080. 10.18544/PEDM-23.02.0080 29073293

[8] Bekx MT, Carrel AL, Shriver TC, Li Z, Allen DB (2003). Decreased energy expenditure is caused by abnormal body composition in infants with Prader-Willi Syndrome. J Pediatr.

[9] Goldstone AP, Holland AJ, Butler JV, Whittington JE (2012). Appetite hormones and the transition to hyperphagia in children with Prader-Willi syndrome. Int J Obes (Lond).

[10] Emerick JE, Vogt KS (2013). Endocrine manifestations and management of Prader-Willi syndrome. Int J Pediatr Endocrinol.

[11] Noordam C, Hoybye C, Eiholzer U. Prader-Willi Syndrome and Hypogonadism: A Review Article. Int J Mol Sci. 2021;22(5). 10.3390/ijms22052705. 10.3390/ijms22052705 PMC7962179 33800122

[12] Pravdivyi I, Ballanyi K, Colmers WF, Wevrick R (2015). Progressive postnatal decline in leptin sensitivity of arcuate hypothalamic neurons in the Magel2-null mouse model of Prader-Willi syndrome. Hum Mol Genet.

[13] Deal CL, Tony M, Hoybye C (2013). GrowthHormone Research Society workshop summary: consensus guidelines for recombinant human growth hormone therapy in Prader-Willi syndrome. J Clin Endocrinol Metab.

[14] Proffitt J, Osann K, McManus B (2019). Contributing factors of mortality in Prader-Willi syndrome. Am J Med Genet A.

[15] Crino A, Grugni G (2020). Update on Diabetes Mellitus and Glucose Metabolism Alterations in Prader-Willi Syndrome. Curr Diab Rep.

[16] Bellis SA, Kuhn I, Adams S, Mullarkey L, Holland A (2022). The consequences of hyperphagia in people with Prader-Willi Syndrome: A systematic review of studies of morbidity and mortality. Eur J Med Genet.

[17] Diene G, Mimoun E, Feigerlova E (2010). Endocrine disorders in children with Prader-Willi syndrome–data from 142 children of the French database. Horm Res Paediatr.

[18] Sinnema M, Maaskant MA, van Schrojenstein Lantman-de Valk HM, et al. Physical health problems in adults with Prader-Willi syndrome. Am J Med Genet A. 2011;155A(9):2112–2124. 10.1002/ajmg.a.34171. 10.1002/ajmg.a.34171 21834028

[19] Miller JL, Linville TD, Dykens EM (2014). Effects of metformin in children and adolescents with Prader-Willi syndrome and early-onset morbid obesity: a pilot study. J Pediatr Endocrinol Metab.

[20] Motaghedi R, Lipman EG, Hogg JE, Christos PJ, Vogiatzi MG, Angulo MA (2011). Psychiatric adverse effects of rimonobant in adults with Prader Willi syndrome. Eur J Med Genet.

[21] Padwal RS, Majumdar SR (2007). Drug treatments for obesity: orlistat, sibutramine, and rimonabant. Lancet.

[22] Hor A, Purtell L.Influence of Naltrexone, Bupropion Combination Treatment on Body Mass Index in Prader-Willi Syndrome Re: "Prader-Willi Syndrome, Management of Impulsivity, and Hyperphagia in an Adolescent" by Puri,  (2016). J Child Adolesc Psychopharmacol.

[23] Fintini D, Grugni G, Brufani C, Bocchini S, Cappa M, Crino A (2014). Use of GLP-1 receptor agonists in Prader-Willi Syndrome: report of six cases. Diabetes Care.

[24] Tan Q, Orsso CE, Deehan EC (2020). Current and emerging therapies for managing hyperphagia and obesity in Prader-Willi syndrome: A narrative review. Obes Rev.

[25] Muscogiuri G, Formoso G, Pugliese G (2019). Prader- Willi syndrome: An uptodate on endocrine and metabolic complications. Rev Endocr Metab Disord.

[26] Schwartz L, Caixas A, Dimitropoulos A (2021). Behavioral features in Prader-Willi syndrome (PWS): consensus paper from the International PWS Clinical Trial Consortium. J Neurodev Disord.

[27] Miller JL, Lynn CH, Driscoll DC (2011). Nutritional phases in Prader-Willi syndrome. Am J Med Genet A.

[28] Damen L, Donze SH, Kuppens RJ (2020). Three years of growth hormone treatment in young adults with Prader-Willi syndrome: sustained positive effects on body composition. Orphanet J Rare Dis.

[29] Lacroix D, Moutel S, Coupaye M (2015). Metabolic and adipose tissue signatures in adults with Prader-Willi syndrome: a model of extreme adiposity. J Clin Endocrinol Metab.

[30] Tanaka Y, Abe Y, Oto Y (2013). Characterization of fat distribution in Prader-Willi syndrome: relationships with adipocytokines and influence of growth hormone treatment. Am J Med Genet A.

[31] Crino A, Fintini D, Bocchini S, Grugni G (2018). Obesity management in Prader-Willi syndrome: current perspectives. Diabetes Metab Syndr Obes.

[32] Hedgeman E, Ulrichsen SP, Carter S (2017). Long-term health outcomes in patients with Prader-Willi Syndrome: a nationwide cohort study in Denmark. Int J Obes (Lond).

[33] Goldstone AP, Holland AJ, Hauffa BP, Hokken-Koelega AC, Tauber M, speakers contributors at the Second Expert Meeting of the Comprehensive Care of Patients with PWS. Recommendations for the diagnosis and management of Prader-Willi syndrome. J Clin Endocrinol Metab. 2008;93(11):4183–4197. 10.1210/jc.2008-0649. 10.1210/jc.2008-0649 18697869

[34] Pellikaan K, Rosenberg AGW, Kattentidt-Mouravieva AA, et al. Missed Diagnoses and Health Problems in Adults With Prader-Willi Syndrome: Recommendations for Screening and Treatment. J Clin Endocrinol Metab. 2020;105(12). 10.1210/clinem/dgaa621. 10.1210/clinem/dgaa621 PMC7553248 32877518

[35] Butler MG, Swift LL, Hill JO (1990). Fasting Plasma Lipid, Glucose, and Insulin Levels in Prader-Willi Syndrome and Obese Individuals. Dysmorphol Clin Genet.

[36] Haqq AM, Muehlbauer MJ, Newgard CB, Grambow S, Freemark M (2011). The metabolic phenotype of Prader-Willi syndrome (PWS) in childhood: heightened insulin sensitivity relative to body mass index. J Clin Endocrinol Metab.

[37] Brambilla P, Crino A, Bedogni G (2011). Metabolic syndrome in children with Prader-Willi syndrome: the effect of obesity. Nutr Metab Cardiovasc Dis.

[38] Manzardo AM, Loker J, Heinemann J, Loker C, Butler MG (2018). Survival trends from the Prader-Willi Syndrome Association (USA) 40-year mortality survey. Genet Med.

[39] Schwingshackl L, Missbach B, Konig J, Hoffmann G (2015). Adherence to a Mediterranean diet and risk of diabetes: a systematic review and meta-analysis. Public Health Nutr.

[40] Butler MG, Theodoro M, Skouse JD (2007). Thyroid function studies in Prader-Willi syndrome. Am J Med Genet A.

[41] Vaiani E, Herzovich V, Chaler E (2010). Thyroid axis dysfunction in patients with Prader-Willi syndrome during the first 2 years of life. Clin Endocrinol (Oxf).

[42] Pellikaan K, Snijders F, Rosenberg AGW, et al. Thyroid Function in Adults with Prader-Willi Syndrome; a Cohort Study and Literature Review. J Clin Med. 2021;10(17). 10.3390/jcm10173804. 10.3390/jcm10173804 PMC8432005 34501256

[43] Festen DA, Visser TJ, Otten BJ, Wit JM, Duivenvoorden HJ, Hokken-Koelega AC (2007). Thyroid hormone levels in children with Prader-Willi syndrome before and during growth hormone treatment. Clin Endocrinol (Oxf).

[44] Di Giorgio G, Grugni G, Fintini D (2014). Growth hormone response to standard provocative stimuli and combined tests in very young children with Prader-Willi syndrome. Horm Res Paediatr.

[45] Bakker NE, Kuppens RJ, Siemensma EP (2013). Eight years of growth hormone treatment in children with Prader-Willi syndrome: maintaining the positive effects. J Clin Endocrinol Metab.

[46] Fillion M, Deal C, Van Vliet G (2009). Retrospective study of the potential benefits and adverse events during growth hormone treatment in children with Prader-Willi syndrome. J Pediatr.

[47] Sode-Carlsen R, Farholt S, Rabben KF (2012). Growth hormone treatment in adults with Prader-Willi syndrome: the Scandinavian study. Endocrine.

[48] Oto Y, Tanaka Y, Abe Y (2014). Exacerbation of BMI after cessation of growth hormone therapy in patients with Prader-Willi syndrome. Am J Med Genet A.

[49] Poitou C, Mosbah H, Clement K (2020). MECHANISMS IN ENDOCRINOLOGY: Update on treatments for patients with genetic obesity. Eur J Endocrinol.

[50] Bellicha A, Coupaye M, Mosbah H, Tauber M, Oppert JM, Poitou C. Physical Activity in Patients with Prader-Willi Syndrome-A Systematic Review of Observational and Interventional Studies. J Clin Med. 2021;10(11). 10.3390/jcm10112528. 10.3390/jcm10112528 PMC8201387 34200339

[51] Butler MG, Theodoro MF, Bittel DC, Donnelly JE (2007). Energy expenditure and physical activity in Prader-Willi syndrome: comparison with obese subjects. Am J Med Genet A.

[52] Castner DM, Tucker JM, Wilson KS, Rubin DA (2014). Patterns of habitual physical activity in youth with and without Prader-Willi Syndrome. Res Dev Disabil.

[53] Bull FC, Al-Ansari SS, Biddle S (2020). World Health Organization 2020 guidelines on physical activity and sedentary behaviour. Br J Sports Med.

[54] Morales JS, Valenzuela PL, Pareja-Galeano H, Rincon-Castanedo C, Rubin DA, Lucia A (2019). Physical exercise and Prader-Willi syndrome: A systematic review. Clin Endocrinol (Oxf).

[55] Grolla E, Andrighetto G, Parmigiani P (2011). Specific treatment of Prader-Willi syndrome through cyclical rehabilitation programmes. Disabil Rehabil.

[56] Silverthorn KH, Hornak JE (1993). Beneficial effects of exercise on aerobic capacity and body composition in adults with Prader-Willi syndrome. Am J Ment Retard.

[57] Kaufman H, Overton G, Leggott J, Clericuzio C (1995). Prader-Willi syndrome: effect of group home placement on obese patients with diabetes. South Med J.

[58] Biro G, Hulshof KF, Ovesen L, Amorim Cruz JA, Group E (2002). Selection of methodology to assess food intake. Eur J Clin Nutr.

[59] Conrad J, Nothlings U (2017). Innovative approaches to estimate individual usual dietary intake in large-scale epidemiological studies. Proc Nutr Soc.

[60] Madden AM, Smith S (2016). Body composition and morphological assessment of nutritional status in adults: a review of anthropometric variables. J Hum Nutr Diet.

[61] Khan SS, Ning H, Wilkins JT (2018). Association of Body Mass Index With Lifetime Risk of Cardiovascular Disease and Compression of Morbidity. JAMA Cardiol.

[62] Ross R, Neeland IJ, Yamashita S (2020). Waist circumference as a vital sign in clinical practice: a Consensus Statement from the IAS and ICCR Working Group on Visceral Obesity. Nat Rev Endocrinol.

[63] Centers for Disease Control and Prevention (CDC). The National Health and Nutrition Examination Survey. Anthropometry Procedures Manual. https://www.cdc.gov/nchs/data/nhanes/nhanes_11_12/Anthropometry_Procedures_Manual.pdf. Accessed 21 Dec 2021.

[64] Riccardi G, Aggett P, Brighenti F, et al. Passclaim--body weight regulation, insulin sensitivity and diabetes risk. Eur J Nutr. 2004;43 Suppl 2:II7-II46. 10.1007/s00394-004-1202-7. 10.1007/s00394-004-1202-7 15221353

[65] Musella M, Milone M, Leongito M, Maietta P, Bianco P, Pisapia A (2014). The mini-gastric bypass in the management of morbid obesity in Prader-Willi syndrome: a viable option?. J Invest Surg.

[66] Smith S, Madden AM (2016). Body composition and functional assessment of nutritional status in adults: a narrative review of imaging, impedance, strength and functional techniques. J Hum Nutr Diet.

[67] Barrea L, Muscogiuri G, Pugliese G, et al. The Sun's Vitamin in Adult Patients Affected by Prader-Willi Syndrome. Nutrients*.* 2020;12(4). 10.3390/nu12041132. 10.3390/nu12041132 PMC7230761 32316673

[68] Woods SG, Knehans A, Arnold S, et al. The associations between diet and physical activity with body composition and walking a timed distance in adults with Prader-Willi syndrome. Food Nutr Res. 2018;62. 10.29219/fnr.v62.1343. 10.29219/fnr.v62.1343 PMC6010474 29942245

[69] Martinez Michel L, Haqq AM, Wismer WV (2016). A review of chemosensory perceptions, food preferences and food-related behaviours in subjects with Prader-Willi Syndrome. Appetite.

[70] Bedogni G, Grugni G, Tringali G, Agosti F, Sartorio A (2015). Assessment of fat-free mass from bioelectrical impedance analysis in obese women with Prader-Willi syndrome. Ann Hum Biol.

[71] Lin HY, Chen MR, Chuang CK, Huang CY, Niu DM, Lin SP (2011). Assessment of body composition using bioelectrical impedance analysis in Prader-Willi syndrome. J Formos Med Assoc.

[72] Hill JO, Kaler M, Spetalnick B, Reed G, Butler MG (1990). Resting Metabolic Rate in Prader-Willi Syndrome. Dysmorphol Clin Genet.

[73] Lazzer S, Grugni G, Tringali G, Sartorio A (2016). Prediction of basal metabolic rate in patients with Prader-Willi syndrome. Eur J Clin Nutr.

[74] Saeed N, Nadeau B, Shannon C, Tincopa M. Evaluation of Dietary Approaches for the Treatment of Non-Alcoholic Fatty Liver Disease: A Systematic Review. Nutrients. 2019;11(12). 10.3390/nu11123064. 10.3390/nu11123064 PMC6950283 31888132

[75] Ditano-Vazquez P, Torres-Pena JD, Galeano-Valle F, et al. The Fluid Aspect of the Mediterranean Diet in the Prevention and Management of Cardiovascular Disease and Diabetes: The Role of Polyphenol Content in Moderate Consumption of Wine and Olive Oil. Nutrients. 2019;11(11). 10.3390/nu11112833. 10.3390/nu11112833 PMC6893438 31752333

[76] Schoeller DA, Levitsky LL, Bandini LG, Dietz WW, Walczak A (1988). Energy expenditure and body composition in Prader-Willi syndrome. Metabolism.

[77] Mach F, Baigent C, Catapano AL (2020). 2019 ESC/EAS Guidelines for the management of dyslipidaemias: lipid modification to reduce cardiovascular risk. Eur Heart J.

[78] Siervo M, Shannon OM, Llewellyn DJ, Stephan BC, Fontana L (2021). Mediterranean diet and cognitive function: From methodology to mechanisms of action. Free Radic Biol Med.

[79] Barrea L, Muscogiuri G, Pugliese G, et al. Phase Angle as an Easy Diagnostic Tool of Meta-Inflammation for the Nutritionist. Nutrients. 2021;13(5). 10.3390/nu13051446. 10.3390/nu13051446 PMC8145306 33923291

[80] Barrea L, Pugliese G, de Alteriis G, Colao A, Savastano S, Muscogiuri G. Phase Angle: Could Be an Easy Tool to Detect Low-Grade Systemic Inflammation in Adults Affected by Prader-Willi Syndrome? Nutrients. 2020;12(7). 10.3390/nu12072065. 10.3390/nu12072065 PMC7400955 32664600

[81] Stobaus N, Pirlich M, Valentini L, Schulzke JD, Norman K (2012). Determinants of bioelectrical phase angle in disease. Br J Nutr.

[82] Butler MG, Bittel DC, Kibiryeva N, Garg U (2006). C-reactive protein levels in subjects with Prader-Willi syndrome and obesity. Genet Med.

[83] Caixas A, Gimenez-Palop O, Broch M (2008). Adult subjects with Prader-Willi syndrome show more low-grade systemic inflammation than matched obese subjects. J Endocrinol Invest.

[84] Hoybye C (2006). Inflammatory markers in adults with Prader-Willi syndrome before and during 12 months growth hormone treatment. Horm Res.

[85] Miller JL, Lynn CH, Shuster J, Driscoll DJ (2013). A reduced-energy intake, well-balanced diet improves weight control in children with Prader-Willi syndrome. J Hum Nutr Diet.

[86] Irizarry KA, Mager DR, Triador L, Muehlbauer MJ, Haqq AM, Freemark M (2019). Hormonal and metabolic effects of carbohydrate restriction in children with Prader-Willi syndrome. Clin Endocrinol (Oxf).

[87] Felix G, Kossoff E, Barron B, Krekel C, Testa EG, Scheimann A (2020). The modified Atkins diet in children with Prader-Willi syndrome. Orphanet J Rare Dis.

[88] Bedogni G, Grugni G, Cicolini S, Caroli D, Tamini S, Sartorio A. Changes of Body Weight and Body Composition in Obese Patients with Prader-Willi Syndrome at 3 and 6 Years of Follow-Up: A Retrospective Cohort Study. J Clin Med. 2020;9(11). 10.3390/jcm9113596. 10.3390/jcm9113596 PMC7695203 33171647

[89] Miller JL, Tan M (2020). Dietary Management for Adolescents with Prader-Willi Syndrome. Adolesc Health Med Ther.

[90] Bonfig W, Dokoupil K, Schmidt H (2009). A special, strict, fat-reduced, and carbohydrate-modified diet leads to marked weight reduction even in overweight adolescents with Prader-Willi syndrome (PWS). ScientificWorldJournal.

[91] Schmidt H, Pozza SB, Bonfig W, Schwarz HP, Dokoupil K (2008). Successful early dietary intervention avoids obesity in patients with Prader-Willi syndrome: a ten-year follow-up. J Pediatr Endocrinol Metab.

[92] Grugni G, Sartorio A, Crino A (2016). Growth hormone therapy for Prader-willi syndrome: challenges and solutions. Ther Clin Risk Manag.

[93] Sipila I, Sintonen H, Hietanen H (2010). Long-term effects of growth hormone therapy on patients with Prader-Willi syndrome. Acta Paediatr.

[94] Butler MG (2006). Management of obesity in Prader-Willi syndrome. Nat Clin Pract Endocrinol Metab.

[95] Consoli A, Cabal Berthoumieu S, Raffin M (2019). Effect of topiramate on eating behaviours in Prader-Willi syndrome: TOPRADER double-blind randomised placebo-controlled study. Transl Psychiatry.

[96] Ali KF, Shukla AP, Aronne LJ (2016). Bupropion-SR plus naltrexone-SR for the treatment of mild-to-moderate obesity. Expert Rev Clin Pharmacol.

[97] Barrea L, Pugliese G, Muscogiuri G, Laudisio D, Colao A, Savastano S. New-generation anti-obesity drugs: naltrexone/bupropion and liraglutide. An update for endocrinologists and nutritionists. Minerva Endocrinol. 2020;45(2):127–137. 10.23736/S0391-1977.20.03179-X. 10.23736/S0391-1977.20.03179-X 32643356

[98] Bueno M, Boixadera-Planas E, Blanco-Hinojo L, et al. Hunger and Satiety Peptides: Is There a Pattern to Classify Patients with Prader-Willi Syndrome? J Clin Med. 2021;10(21). 10.3390/jcm10215170. 10.3390/jcm10215170 PMC8585040 34768690

[99] Cheang JY, Moyle PM (2018). Glucagon-Like Peptide-1 (GLP-1)-Based Therapeutics: Current Status and Future Opportunities beyond Type 2 Diabetes. ChemMedChem.

[100] Salehi P, Hsu I, Azen CG, Mittelman SD, Geffner ME, Jeandron D (2017). Effects of exenatide on weight and appetite in overweight adolescents and young adults with Prader-Willi syndrome. Pediatr Obes.

[101] Allas S, Caixas A, Poitou C (2018). AZP-531, an unacylated ghrelin analog, improves food-related behavior in patients with Prader-Willi syndrome: A randomized placebo-controlled trial. PLoS ONE.

[102] Tauber M, Diene G (2021). Prader-Willi syndrome: Hormone therapies. Handb Clin Neurol.

[103] Tripodi M, Casertano A, Peluso M (2020). Prader-Willi Syndrome: Role of Bariatric Surgery in Two Adolescents with Obesity. Obes Surg.

[104] Scheimann AO, Butler MG, Gourash L, Cuffari C, Klish W (2008). Critical analysis of bariatric procedures in Prader-Willi syndrome. J Pediatr Gastroenterol Nutr.

[105] Alqahtani AR, Elahmedi MO, Al Qahtani AR, Lee J, Butler MG (2016). Laparoscopic sleeve gastrectomy in children and adolescents with Prader-Willi syndrome: a matched-control study. Surg Obes Relat Dis.

[106] Fong AK, Wong SK, Lam CC, Ng EK (2012). Ghrelin level and weight loss after laparoscopic sleeve gastrectomy and gastric mini-bypass for Prader-Willi syndrome in Chinese. Obes Surg.

[107] Scheimann AO, Miller J, Glaze DG (2017). Laparoscopic sleeve gastrectomy in children and adolescents with Prader-Willi syndrome: a matched control study. Surg Obes Relat Dis.

[108] Liu SY, Wong SK, Lam CC, Ng EK (2020). Bariatric surgery for Prader-Willi syndrome was ineffective in producing sustainable weight loss: Long term results for up to 10 years. Pediatr Obes.

[109] Di Pietro ML, Zace D (2020). Three scenarios illustrating ethical concerns when considering bariatric surgery in obese adolescents with Prader-Willi syndrome. J Med Ethics.

